# TRAIL sensitisation by arsenic trioxide is caspase-8 dependent and involves modulation of death receptor components and Akt

**DOI:** 10.1038/sj.bjc.6602954

**Published:** 2006-01-24

**Authors:** E Szegezdi, S Cahill, M Meyer, M O'Dwyer, A Samali

**Affiliations:** 1 Department of Biochemistry, National University of Ireland,University Road, Galway, Ireland; 2 The National Centre for Biomedical Engineering Science, National University of Ireland, University Road, Galway, Ireland; 3 Department of Haematology, University College Hospital Galway, Newcastle Road, Galway, Ireland

**Keywords:** arsenic trioxide, TRAIL, Akt, leukaemia, DR5, FLIP

## Abstract

The majority of leukaemic cells are resistant to apoptosis induced by tumour necrosis factor-related apoptosis-inducing ligand (TRAIL). Here, we show that sublethal concentrations of arsenic trioxide (ATO) specifically enhanced TRAIL-induced apoptosis in leukaemic but not in other tumour cell lines. The combination of ATO and TRAIL synergistically enhanced cleavage of caspase-8, which was blocked by the caspase inhibitor IETD.fmk as well as in cells deficient for caspase-8, suggesting a requirement for the death-inducing signalling complex. Arsenic trioxide led to increased cell surface expression of DR5 (death receptor 5), inhibition of the serine/threonine kinase Akt and downregulation of the short isoform of FLIP (FLICE-inhibitory protein, FLIP_S_). Inhibition of the phosphatidylinositol 3 kinase (PI3K) was equally efficient in sensitising leukaemic cells to TRAIL with similar effects on DR5 and FLIP_S_ expression, suggesting that ATO may in part act through inhibition of the PI3K/Akt signalling pathway. These results indicate that the enhancement in TRAIL-mediated apoptosis induced by ATO is due to alteration in the levels of multiple components and regulators of the death receptor-mediated pathway. These findings offer a promising and novel strategy involving a combination of TRAIL and ATO, or more specific Akt inhibitors in the treatment of various haematopoietic malignancies.

The outcome of adult patients with acute leukaemia has changed little over the past 20 years, especially in older patients (>60 years) in whom intensive chemotherapy is frequently inappropriate and survival poor. Development of less-toxic, targeted approaches is clearly necessary. One such emerging anticancer agent is the tumour necrosis factor-related apoptosis-inducing ligand (TRAIL). Tumour necrosis factor-related apoptosis-inducing ligand is a member of the TNF death ligand family, which can interact with four distinct membrane-bound receptors (DR4/TRAIL-R1, DR5/TRAIL-R2, DcR1/TRAIL-R3 and DcR2/TRAIL-R4) (Wiley *et al*, 1995; Ashkenazi and Dixit, 1999). DR4 and DR5 contain a conserved cytoplasmic region called the death domain (DD) that is required for TRAIL-induced apoptosis. DcR1 does not have a cytoplasmic domain, whereas DcR2 contains a truncated DD motif (Ashkenazi and Dixit, 1999). Thus, DcR1 and DcR2 act as decoy receptors, and ligation of only DR4 or DR5 initiates apoptosis.

Binding of TRAIL to DR4 or DR5 leads to receptor trimerisation and assembly of the death-inducing signalling complex (DISC). Caspase-8 is recruited to the DISC where it becomes activated (Ashkenazi and Dixit, 1999; MacFarlane, 2003). Subsequent events can follow two apoptotic-signalling pathways. In the first scenario (type I cells), caspase-8 directly activates effector caspases (caspase-3, -6, -7), while in type II cells, caspase-8 engages the mitochondrial pathway by activating Bid (Sartorius *et al*, 2001). Tumour necrosis factor-related apoptosis-inducing ligand is an attractive antineoplastic agent as it preferentially induces apoptosis in transformed cells, with little effect on normal cells (Lawrence *et al*, 2001; Almasan and Ashkenazi, 2003). Additionally, TRAIL may overcome drug resistance due to dysfunction of the p53 pathway or overexpression of Bcl-2 family members (Walczak *et al*, 2000; Nagane *et al*, 2001). However, response to TRAIL is highly variable with resistance seen in many cancer types, including leukaemia (Ehrhardt *et al*, 2003). Potential mechanisms of resistance include poor expression of DR4 and/or DR5 or increased expression of decoy receptors and c-FLIP (FLICE-inhibitory protein) (Sheridan *et al*, 1997; Jin *et al*, 2004; Ricci *et al*, 2004). In type II cells due to the requirement for intrinsic pathway amplification, TRAIL resistance also can be mediated at the level of the mitochondria by Bcl-2 and Bcl-X_L_ (Korsmeyer, 1992). Finally, overexpression of inhibitor of apoptosis proteins (IAP) can prevent TRAIL-induced apoptosis through inhibition of effector caspases (caspase-3, -6 and -7) (Aggarwal *et al*, 2004). Notably, the expression of these different antiapoptotic proteins may be regulated by several different signal transduction pathways including Akt, NF-*κ*B and p53, which are frequently activated in leukaemias (Shankar and Srivastava, 2004). Strategies to overcome these mechanisms of resistance are the subject of intensive investigation. A variety of anticancer modalities including cytotoxic chemotherapy, radiation and novel therapies were shown to have additive or synergistic effects with TRAIL (Sayers *et al*, 2003; Ganten *et al*, 2004; Inoue *et al*, 2004; Rosato *et al*, 2004). However, in most of these studies, no mechanistic insight was provided.

Based on a recent report demonstrating synergy between arsenic trioxide (ATO) and TRAIL in myeloma cells, we were interested in exploring the potential of ATO as a TRAIL-sensitising agent in leukaemic cells (Liu *et al*, 2003). Arsenic trioxide has pleiotropic mechanisms of action. Differential effects with respect to induction of differentiation and apoptosis have been described, which appear to be dose dependent (Chen *et al*, 1996). At low, clinically achievable concentration, ATO has demonstrated marked activity in acute promyelocytic leukaemia (APL) with little toxicity (Niu *et al*, 1999; O'Dwyer *et al*, 2002). In other haematological malignancies however, as a single agent, ATO has only a modest activity, although it may have a greater role in combination with other drugs (Evens *et al*, 2004).

We examined a selection of tumour cell lines, (including three leukemic cell lines) looking for evidence of synergy between ATO and TRAIL and specifically, to identify the cellular mechanisms underlying this effect. In all three leukaemic cell lines tested, ATO augmented TRAIL-induced cell death. This could be blocked by inhibition or absence of caspase-8 suggesting a requirement for the DISC complex. Arsenic trioxide increased cell surface expression of DR5, inhibited phosphorylation of Akt and led to downregulation of the short isoform of FLIP (FLIP_S_). Inhibition of the PI3K was equally efficient in sensitising leukaemic cells to TRAIL with similar effects on DR5 and FLIP_S_ expression, suggesting that ATO may act through inhibition of the PI3K/Akt signalling pathway. These results point out that the combination of TRAIL and ATO, or more specific Akt inhibitors may be valuable in the treatment of various haematopoietic malignancies.

## MATERIALS AND METHODS

### Cell culture and treatments

Jurkat, caspase-8-deficient Jurkat (Juo *et al*, 1998), ML-1, K562, Raji and Colo205 cells were cultured in RPMI medium. PC3, HeLa, cells were cultured in DMEM medium. Both media were supplemented with 10% foetal bovine serum, 2 mM glutamine, 50 U ml^−1^ penicillin and 5 mg ml^−1^ streptomycin, RPMI medium was also supplemented with 1 mM Na-pyruvate. To induce apoptosis, cells were treated with various concentrations of recombinant TRAIL (soluble TRAIL (aa 114–281) was a kind gift from Professor Wim J Quax, University of Groningen, Groningen, the Netherlands) in the presence or absence of increasing concentration of ATO (100–1000 ng ml^−1^, 0.5–5 *μ*M) for the indicated time periods. The PI3K inhibitor LY294002 (Calbiochem, Schwalbach, Germany) and the caspase inhibitor IETD.fmk (Enzyme Systems Products, Livermore, CA, USA) were added 1 h prior to treatment with TRAIL at a final concentration of 20 *μ*M. All other reagents were from Sigma-Aldrich, St Louis, MO, USA, unless indicated otherwise.

### Western blot analysis

Cells were lysed in a buffer containing 20 mM HEPES, pH 7.5, 350 mM NaCl, 1 mM MgCl_2_, 0.5 mM EDTA, 0.5 mM EGTA, 1% NP-40, 0.5 mM dithiothreitol (DTT), 100 *μ*M PMSF (phenylmethanesulphonyl fluoride), 2 *μ*g ml^−1^ pepstatin A, 25 *μ*M ALLN, 2.5 *μ*g ml^−1^ aprotinin and 10 *μ*M leupeptin. Proteins were separated by electrophoresis on 10% SDS–polyacrylamide gels and then transferred onto nitrocellulose membranes. After blocking in 5% nonfat milk, 0.05% Tween-20 in PBS, blots were incubated with 1 : 1000 dilution of antibodies to caspase-8, P-Akt (Ser 473), Akt (all from Cell Signaling Technology, Danvers, MA, USA), c-FLIP (Stressgen, Victoria, BC, Canada) and 1 : 500 dilution of rabbit polyclonal antibody against *β*-actin (Sigma, St Louis, MO, USA). The appropriate horseradish peroxidase-conjugated goat secondary antibodies (Pierce, Rockford, IL, USA) were used at a 1 : 5000 for primary antibodies from Cell Signaling Technologies and at a 1 : 10 000 dilution for all other antibodies. Protein bands were detected with Super Signal Ultra Chemilumiescent Substrate (Pierce) on X-ray film (Agfa, Mortsel, Belgium).

### Haematoxylin–Eosin staining and microscopy

After treatments, cells were spun onto microscope slides, fixed in methanol for 5 min at room temperature and were stained by immersion in Harris haematoxylin solution for 5 min followed by 1 min immersion in Eosin Y. The slides were mounted using DPX. Phase contrast images were taken from 15 randomly chosen areas per sample using 400 × overall magnification (Zeiss S100 Microscope). For data acquisition, the AQM acquisition manager program was used.

### Caspase activity assay

Cells were harvested by centrifuging at 450 *g* for 5 min. After two washes in PBS, duplicate samples of 25 *μ*l were transferred to a microtitre plate and snap-frozen over liquid nitrogen. To initiate the reaction, 50 *μ*M of the caspase substrate Ac-Ile-Glu-Thr-Asp-*α*-(4-methyl-coumaryl-7-amide) (IETD-AMC) (Peptide Institute Inc., Osaka, Japan) in assay buffer (100 mM HEPES, 10% sucrose, 5 mM DTT, 0.0001% NP-40 and 0.1% 3-[(3-cholamidopropyl) dimethylammonio] propane-1-sulphonic acid (CHAPS), pH 7.25) was added to the cell lysates. Liberated AMC was measured at 37°C kinetically every 60 s, for 25 repeats on a Wallac Victor^2^ plate reader using excitation and emission wavelengths of 355 and 460 nm, respectively. Enzyme activity was expressed as nanomole AMC released per minute per mg of protein.

### MTT assay

MTT dye (250 *μ*g ml^−1^) was added to control and treated cells and incubated for 3 h at 37°C. The reaction was stopped and the blue formazan precipitate formed was dissolved using 20% SDS in 50% dimethylformamide. The colour intensity was measured at 550 nm on a Wallac Victor^2^ plate reader. The control value corresponding to untreated cells was taken as 100% and the viability of treated samples was expressed as a percentage of the control.

### Apoptosis detection assay

Cells were treated with TRAIL and ATO for 6–12 h and PS (phosphatidyl serine) exposure was measured using annexin-V-FITC (IQ Corporation, Groningen, The Netherlands) as described earlier (Concannon *et al*, 2005). Briefly, cells were collected by centrifugation at 350 *g*, washed once in ice-cold calcium buffer (10 mM HEPES/NaOH, pH 7.4, 140 mM NaCl, 2.5 mM CaCl_2_) and incubated with annexin V-FITC for 15 min on ice. A wash step in calcium buffer was carried out prior to acquisition on a FacsCalibur flow cytometer (Becton Dickinson, Franklin Lakes, NJ, USA).

### Cell surface expression of TRAIL receptors

Cells were washed twice in PBS containing 1% BSA and then incubated with monoclonal antibodies to DR4, DR5, DcR1 and DcR2 (Alexis, Lausen, Switzerland) for 40 min. After two wash steps with PBS/BSA, anti-mouse IgG-FITC (Sigma) secondary antibody was added for 30 min. All incubations were carried out at room temperature. Negative controls contained only secondary antibody. Cell surface expression was analysed by a FacsCalibur flow cytometer.

## RESULTS

### Synergistic cytotoxic effect of TRAIL and ATO on leukaemic cells

We examined whether treatment with sublethal doses of ATO can sensitise tumour cells to TRAIL-induced apoptosis. A panel of seven human cancer cell lines was tested, including three adenocarcinomas, a B-cell lymphoma and three leukaemias. Treatment with TRAIL alone revealed varying sensitivity between the different cell types. The most TRAIL-sensitive of the seven cell lines was the Colo205 colon adenocarcinoma cell line, where 10 ng ml^−1^ TRAIL caused almost 100% cell death within 24 h. The myeloid leukaemia ML-1 and the prostate adenocarcinoma PC3 cells were also sensitive but required 200 ng ml^−1^ TRAIL over 24 h for 90% cell death. In the remaining cell lines, TRAIL could not induce more than 20% cell death over 24 h even at the highest concentration used (1.25 *μ*g ml^−1^) (Table 1).

**Figure 1:**
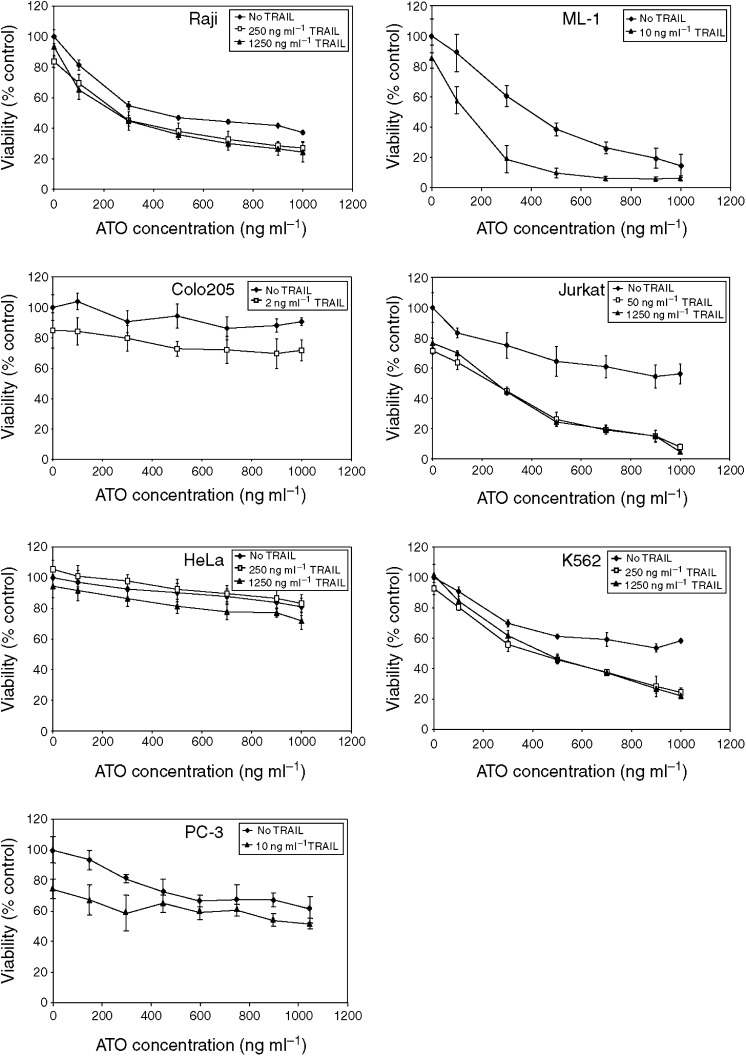
Tumour necrosis factor-related apoptosis-inducing ligand and ATO has synergistic cytotoxic effect in leukaemic cells. Seven tumour cell lines were treated with TRAIL in the presence of increasing concentration of ATO for 24 h. Cell viability was measured by MTT assay. The graphs show the average cell viability ±s.d. from three independent experiments expressed as percentage of untreated cells.

**Figure 2:**
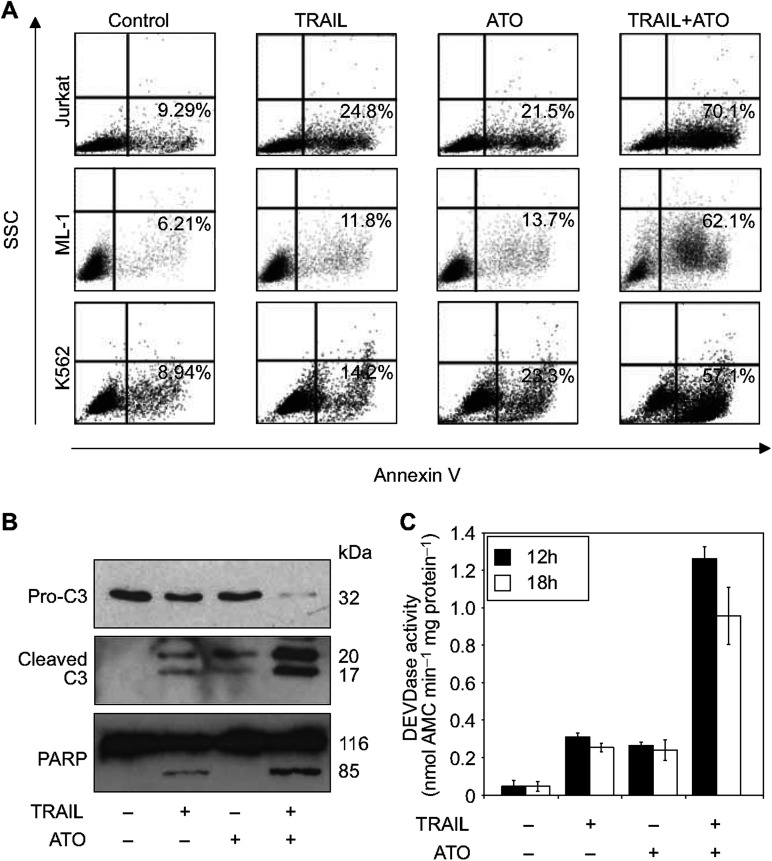
Combined treatment with TRAIL and ATO has synergistic apoptosis-inducing effect. Cells were treated with sublethal dose of TRAIL (Jurkat: 50 ng ml^−1^, ML-1: 10 ng ml^−1^, K562: 250 ng ml^−1^) in the presence or absence of ATO (500 ng ml^−1^). (**A**) Apoptotic cell death was measured 12 h post treatment by annexin V staining. One representative dot plot image from three independent experiments is shown. (**B**) Western blot analysis of pro-caspase-3 and PARP cleavage in Jurkat cells treated with TRAIL and ATO for 10 h. The figure shows one representative picture of three independent experiments. (**C**) DEVDase activity in TRAIL- and ATO-treated Jurkat cells. DEVDase activity was measured in whole-cell lysates with a kinetic assay. Enzyme activity was expressed in nmole AMC released per minute by 1 mg total cellular protein. The chart represents the result of three independent experiments ±s.d.

To test the effect of ATO on TRAIL sensitivity of these tumour cells, for each cell line the lowest TRAIL concentration that caused 10–20% cell death was determined. Using this TRAIL concentration, the cells were treated with increasing concentration of ATO (100–1000 ng ml^−1^, i.e. 0.5–5 *μ*M) for 24 h and cell viability was determined with MTT assay (Figure 1). In case of the TRAIL-resistant cell lines, the highest TRAIL concentration (1.25 *μ*g ml^−1^) was also tested, but no difference was observed (Figure 1). Of the seven cell lines, none of the adenocarcinomas responded to ATO treatment with increased TRAIL sensitivity (Table 1). However, all three leukaemia cell lines (Jurkat, ML-1 and K562) showed enhanced cell death in response to the combined treatment. Phosphatidyl serine exposure measured by the extent of annexin V binding confirmed the synergistic death-inducing effect of TRAIL and ATO in the three leukaemic cell lines. In Jurkat cells, 12 h treatment with TRAIL or ATO induced 15% and 12% annexin V positivity, respectively. However, the combined treatments led to 69% positivity in 12 h. Similar results were obtained in ML-1 and K562 cells (Figure 2A). Cell death induced by the combined treatment displayed features of apoptosis, including blebbing, nuclear condensation (data not shown), pro-caspase-3 processing and activation, as well as cleavage of the caspase-3 substrate protein PARP (Figure 2B and C).

### Sensitisation by ATO is caspase-8 dependent

**Figure 3:**
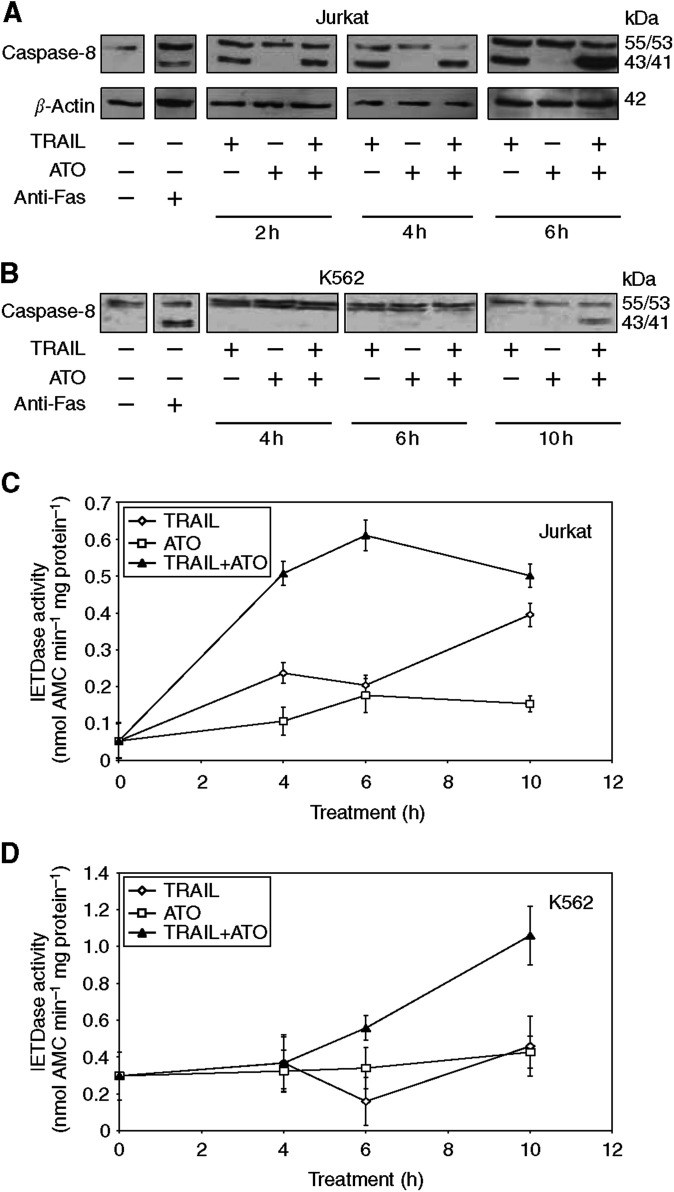
Combined TRAIL and ATO treatment leads to increased pro-caspase-8 cleavage and activation. Jurkat and K562 cells were treated with TRAIL (50 and 250 ng ml^−1^, respectively), ATO (500 ng ml^−1^) or with both for the indicated times. Pro-caspase-8 cleavage (**A**, **B**) and IETDase activity (**C**, **D**) were measured in Jurkat (**A**, **C**) and K562 (**B**, **D**) cell lysates. In part A, *β*-actin was used as a loading control. Enzyme activity was expressed as nmole AMC released per minute per mg total cellular protein. The graphs show the average ±s.d. of three independent experiments.

To delineate the mechanism by which low-dose ATO modifies sensitivity to TRAIL, activation of pro-caspase-8 in Jurkat and K562 cells was examined by following pro-caspase-8 cleavage with Western blotting (Figure 3A and B) and by a fluorescent IETDase assay (Figure 3C and D). In Jurkat cells, treatment with TRAIL alone induced some pro-caspase-8 processing, which was detectable after 2 h treatment (Figure 3A). Longer incubation with TRAIL led only to a marginal increase in pro-caspase-8 processing. Combined treatment with TRAIL and ATO induced pro-caspase-8 processing similar to TRAIL alone at 2 h; however, as the incubation time increased, a much more pronounced, almost complete processing of pro-caspase-8 was detectable (Figure 3A). In addition to the p43/p41 cleavage product, the fully processed p18 caspase-8 subunit was also detectable in the combined treatments (data not shown), indicating the lack of c-FLIP_L_-mediated inhibition of caspase-8 (Krueger *et al*, 2001). Measurement of IETDase activity reflected the same pattern with high enzyme activity detectable only in the combination treatment peaking at 6 h of treatment (Figure 3C). In K562 cells, TRAIL or ATO alone failed to induce detectable pro-caspase-8 processing or activity over the 10 h incubation time (Figure 3B and D). Combined treatment with TRAIL and ATO for 10 h, however, induced pro-caspase-8 cleavage and a pronounced IETDase activity.

**Figure 4:**
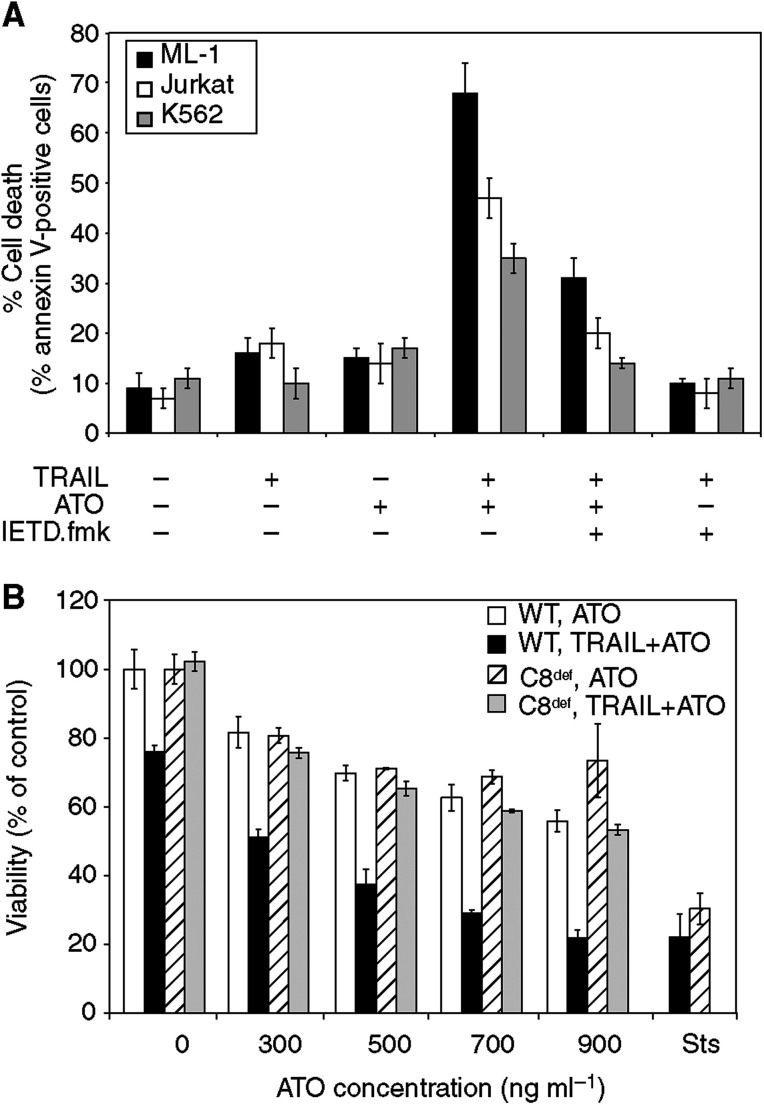
Inhibition or deficiency of caspase-8 prevents cell death induced by combined TRAIL+ATO treatment. (**A**) Cells were treated with TRAIL (Jurkat: 50 ng ml^−1^, ML-1: 10 ng ml^−1^, K562: 250 ng ml^−1^), ATO (500 ng ml^−1^) or both in the presence or absence of IETD.fmk (20 *μ*M). Cell death was measured by flow cytometry after annexin V staining. Results are represented as average cell death ±s.d. of three independent experiments. (**B**) Caspase-8-expressing (WT) and caspase-8-deficient (C8^def^) Jurkat cells were treated with increasing doses of ATO with or without TRAIL (50 ng ml^−1^). Viability was measured by MTT assay. The graph shows the average of two independent experiments ±s.d.

In order to examine the requirement of caspase-8 in the process, we used two approaches. Prior to treatment with TRAIL and ATO, ML-1, K562 and Jurkat cells were treated with the caspase inhibitor IETD.fmk, which is thought to preferentially target caspase-8 and -10. Pretreatment with IETD.fmk partially prevented the synergistic action of TRAIL and ATO in all the three cell lines tested (Figure 4A). In the second approach, we used a caspase-8-deficient Jurkat cell line. In these cells, 24 h treatment with ATO had minor cytotoxic effect, similar to that found in wild-type Jurkat cells. Addition of TRAIL in combination with ATO however, caused no increase in cell death, providing compelling evidence for a caspase-8-dependent action of the combined TRAIL and ATO treatment (Figure 4B). Requirement for caspase-8 further proves that ATO, at the concentrations used, primarily acts to reactivate the TRAIL death pathway, not as a TRAIL-independent cytotoxic stimulus.

### ATO increases DR5 cell surface expression

**Figure 5:**
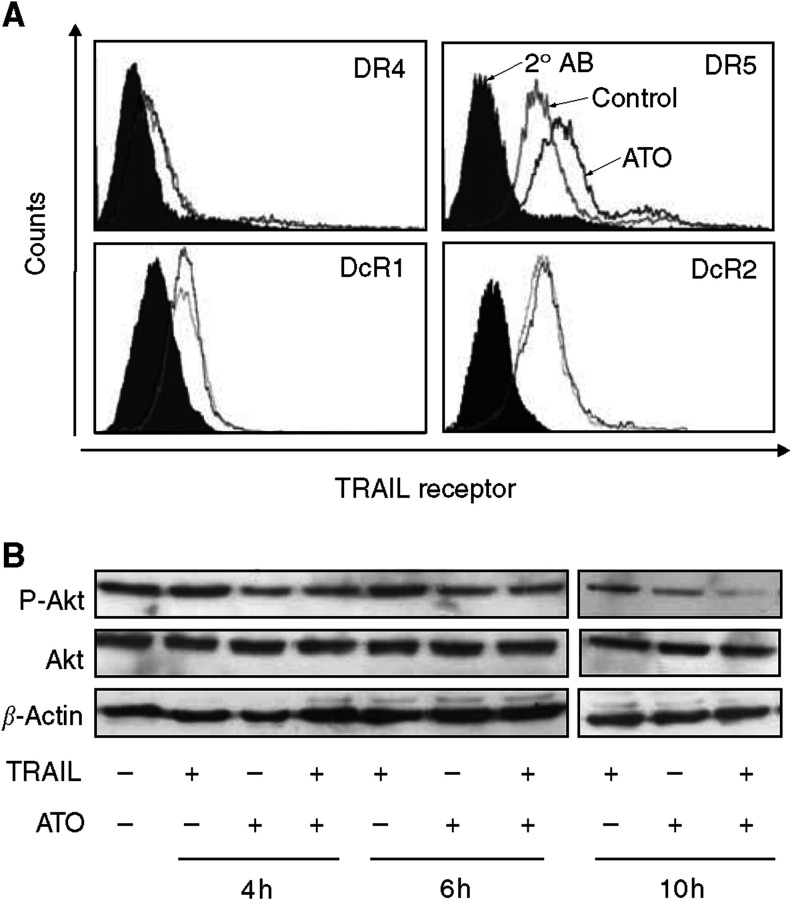
Aarsenic trioxide affects Akt phosphorylation and DR5 cell surface expression. (**A**) Flow cytometric analysis of cell surface expression of TRAIL receptors DR4, DR5, DcR1 and DcR2 in Jurkat cells after treatment with 500 ng ml^−1^ ATO for 6 h. (**B**) Western blot analysis of phosphorylated Akt and total cellular Akt in Jurkat cells treated with 50 ng ml^−1^ TRAIL in the presence or absence of 500 ng ml^−1^ ATO for the indicated times. *β*-Actin was used as a loading control. In all cases, one representative image of three independent experiments is shown.

As TRAIL and ATO enhanced caspase-8 activation and their synergistic action was caspase-8 dependent, it prompted us to identify whether components of the DISC complex are affected by ATO. The cell surface expression of DR4, DR5 and the two decoy receptors, DcR1 and DcR2, was assessed in Jurkat cells. Although there was no change in the expression of the two decoy receptors and DR4, the cell surface expression of DR5 was increased after 6 h treatment with ATO (Figure 5A).

### ATO affects phosphorylation state of Akt

Akt is a serine/threonine kinase, a target of the phosphatidylinositol 3 kinase (Brazil *et al*, 2002). It has a strong antiapoptotic and proliferative function and is known to be activated (phosphorylated) in several leukaemias. Jurkat cells also have considerable basal Akt activity. Examination of the effect of ATO on Akt phosphorylation in Jurkat cells by Western blotting revealed that ATO treatment resulted in a rapid (4–6 h) and significant decrease in Akt phosphorylation. The decrease in the level of phosphorylated Akt (P-Akt) was due to dephosphorylation of Akt, rather then a reduction in total Akt protein levels (Figure 5B).

**Figure 6:**
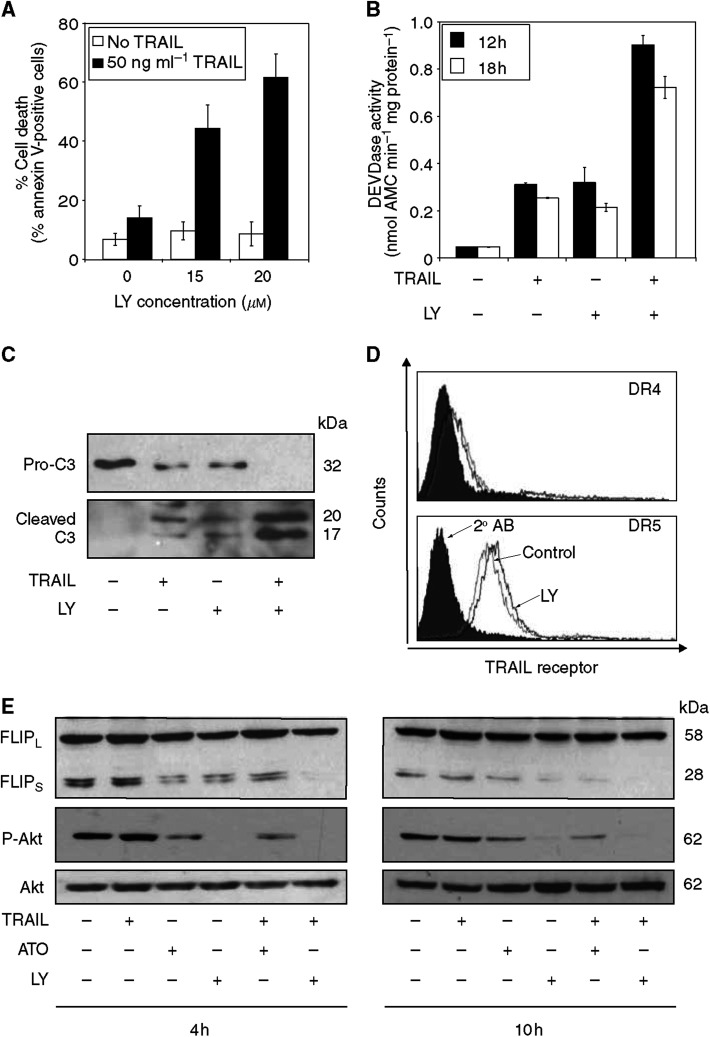
Inhibition of Akt signalling mimics the effect of ATO in Jurkat cells. Cells were treated with the PI3K inhibitor LY294002 (20 *μ*M) 1 h prior to treatment with TRAIL for 12 h. Cell death was assessed by FACS analysis of annexin V-stained cells. The graph shows the average values of three independent experiments ±s.d. (**B**) DEVDase activity in cells treated with TRAIL, ATO or both for the indicated times. DEVDase activity was measured in whole-cell lysates with a kinetic assay. Enzyme activity was expressed as nmole AMC released per minute by 1 mg total cellular protein. The chart represents the result of three independent experiments ±s.d. (**C**) Western blot analysis of pro-caspase-3 cleavage in lysates of Jurkat cells treated with TRAIL (50 ng ml^−1^), ATO (500 ng ml^−1^) and TRAIL+ATO for 10 h. The figure shows one representative picture from three independent experiments. (**D**) FACS analysis of DR4 and DR5 cell surface expression after 5 h treatment with LY294002 (20 *μ*M). The histograms are representatives of at least three independent experiments. (**E**) Western blot analysis of phosphorylated Akt, Akt and c-FLIP following treatment with 20 *μ*M LY294002 or 500 ng ml^−1^ ATO in the presence or absence of 50 ng ml^−1^ TRAIL for the times indicated. The figure is one representative of three independent experiments.

In order to address the possible link between Akt and TRAIL sensitivity, ATO was replaced with the PI3K inhibitor LY294002 (20 *μ*M) in the combination treatment with TRAIL. Pretreatment of cells with LY294002 for 1 h also led to increased TRAIL responsiveness. Cell death measured by annexin V staining (Figure 6A) synergistically increased after combined treatment and cells showed features of apoptosis, including typical morphological changes (data not shown), pro-caspase-3 processing and activation (Figure 6B and C). Akt phosphorylation was also examined after treatment with the PI3K inhibitor LY294002 in order to compare the Akt inhibiting potential of ATO and LY294002. Treatment with LY294002 caused rapid and complete dephosphorylation of Akt in 4 h (Figure 6E). Arsenic trioxide also induced similar, although not complete, inhibition of Akt after 4 h (Figure 6E).

Since treatment with ATO increased cell surface expression of DR5, we examined if treatment with LY294002 mimicked this effect. A treatment of Jurkat cells with LY294002 for 4 h, although to a lesser extent, also increased cell surface expression of DR5, without affecting DR4 (Figure 6D). A possible alteration in c-FLIP, a downstream target of Akt and a common inhibitor of TRAIL-induced apoptosis was also examined (Figure 6E). We found that treatment with either ATO or LY294002 caused a rapid and pronounced drop in the expression of the short form of c-FLIP, detectable after 4 h treatment, pointing to a possible common mechanism of action. Together, these data suggest that the mechanism by which ATO enhances TRAIL sensitivity involves inhibition of Akt and primarily targets components of the DR5 DISC complex.

## DISCUSSION

Elemental arsenic has been used for centuries in medication and arsenic was the first successful treatment for CML. Recently, the trioxide derivative of arsenic has been shown to have marked activity against relapsed APL inducing complete remission in 87% of patients with only minimal side effects (Soignet *et al*, 2001; Douer and Tallman, 2005). The multiple mechanisms of action of ATO suggest that it may have antitumour activity in malignancies other than APL and that it may be used in combination with other agents. In the present study, we examined the mechanism of ATO-mediated sensitisation of cancer cells to TRAIL-induced apoptosis. We found that ATO specifically enhanced TRAIL-mediated cell death in leukaemic cells, but not in other cancer cell types. Our results indicate that the mechanism involves inhibition of the PI3K/Akt pathway, and modulation of the TRAIL receptor DISC components FLIP_S_ and DR5.

Studying the mechanism of ATO-mediated sensitisation revealed that combined treatment of Jurkat and K562 cells with TRAIL and ATO led to increased processing and activation of pro-caspase-8. The synergistic apoptosis-inducing effect appeared to be caspase-8 dependent, as IETD.fmk-treated and caspase-8-deficient cells were resistant to combined TRAIL and ATO-induced apoptosis. As in most haematopoietic cells, TRAIL induces the type I pathway, that is, mitochondrial amplification is not required, and our findings suggested that the synergistic effect is at the level of the DISC. To understand this effect further, we examined the components of the DISC and observed early upregulation of DR5 along with downregulation of c-FLIP_S_. Upregulation of DR5 has been reported following treatment of different cancer cell lines with a variety of anticancer agents, where both p53-dependent and -independent mechanisms are recognised (Wang and El-Deiry, 2003; Insinga *et al*, 2005). Following treatment with ATO, myeloma cell lines with varying p53 status, all showed upregulation of DR4 and DR5 and synergy with TRAIL (Akay and Gazitt, 2003; Liu *et al*, 2003). Since in Jurkat cells p53 is mutated, our data confirm that p53 is not required for ATO-mediated surface induction of DR5 (Cheng and Haas, 1990; Trepel *et al*, 1997).

The observed decline in c-FLIP_S_ levels following ATO is particularly interesting. c-FLIP exists in various isoforms, the best-characterised are c-FLIP long (c-FLIP_L_) and c-FLIP short (c-FLIP_S_). c-FLIP has homology to caspases-8, and is able to inhibit apoptosis by acting as a competitive antagonist of caspase-8 (Irmler *et al*, 1997). Overexpression and knock down studies as well as reported correlations between c-FLIP levels and reduced sensitivity to apoptosis implicate c-FLIP as a key inhibitor of death receptor-induced apoptosis (Ganten *et al*, 2004; Sharp *et al*, 2005). In Jurkat cells, ATO treatment caused a pronounced decrease in c-FLIP_S_ expression, detectable very early, after 4 h treatment. Decreased c-FLIP levels together with increased DR5 cell surface expression could lead to an increased pro-caspase-8 to c-FLIP ratio in the DISC resulting in enhanced pro-caspase-8 activation and eventually cell death. In line with this hypothesis, we detected a strong increase in pro-caspase-8 cleavage and activity after 4 and 6 h treatment with TRAIL and ATO.

We also demonstrated that ATO treatment resulted in inhibition of the serine/threonine kinase Akt (Brazil *et al*, 2002). Furthermore, blocking the PI3K-mediated activation of Akt led to similar effects to that seen with ATO including DR5 upregulation, c-FLIP_S_ downregulation and greatly increased responsiveness to TRAIL. From these observations we conclude that inhibition of Akt is sufficient to restore TRAIL sensitivity, and the TRAIL-sensitising effect of ATO is at least in part due to inhibition of Akt. Akt is frequently activated in various cancers and moreover, high levels of phosphorylated (activated) Akt is a poor prognostic indicator in CML (Vivanco and Sawyers, 2002; Xu *et al*, 2003; Thompson and Thompson, 2004; Grandage *et al*, 2005). To promote cell survival, Akt phoshorylates and inhibits the pro-apoptotic protein BAD, downregulates the expression of Bim and at the same time upregulates antiapoptotic proteins, such as c-FLIP, IAPs (inhibitor of apoptosis proteins) and Bcl-2 by activating NF-*κ*B (Thompson and Thompson, 2004). Akt is also known to mediate TRAIL resistance (Nam *et al*, 2003). A constitutively activated Akt protected HL-60 cells (an APL cell line) from TRAIL-induced apoptosis through a mechanism involving NF-*κ*B activation and c-FLIP_L_ upregulation, all of which could be reversed by treatment with PI3K inhibitors (Bortul *et al*, 2003). These findings are similar to our own observations, although the authors of this report did not find any change in cell surface expression of death receptors, highlighting that the response to Akt inhibition is likely to be cell type specific.

As yet, we cannot rule out that other mechanisms also contribute to the TRAIL-potentiating effect of ATO. For example, ATO is a potent inducer of histone hyperacetylation (Perkins *et al*, 2000; Chen *et al*, 2001). While the present manuscript was in preparation, GM Cohen's lab published that histone deacetylase inhibitors, in very low concentrations, are able to sensitise TRAIL-resistant chronic lymphocytic leukaemia cells (Inoue *et al*, 2004). The mechanism they revealed involves DR5 upregulation, facilitated TRAIL receptor DISC formation and enhanced caspase-8 processing, many features similar to our findings.

In summary, our data provide for the first time a potential new usage of ATO, that is, in combination with TRAIL as an antieukemic agent. At low, easily achievable concentrations, ATO can reverse TRAIL resistance, probably in part due to inhibition of Akt. While Akt inhibitors are not yet clinically available, our results suggest that ATO, an already approved medication, can achieve the same effect and offers a combination therapy with TRAIL as a novel systemic therapy for leukaemias.

## Figures and Tables

**Table 1:** Semiquantitative representation of TRAIL and ATO sensitivity of tested cell lines

**Cell line**	**Origin**	**TRAIL sensitivity**	**Synergism with ATO**
PC3	Prostate adenocarcinoma	+++	+
HeLa	Cervical adenocarcinoma	+	+
Colo205	Colon adenocarcinoma	+++++	+
Raji	Burkitt's lymphoma	+	+
ML-1	Acute myeloid leukaemia	+++	+++++
K562	Chronic myelogenous leukemia	+	+++++
Jurkat	Acute T-cell leukaemia	++	+++++
